# Using phenomic selection to predict hybrid values with NIR spectra measured on the parental lines: proof of concept on maize

**DOI:** 10.1007/s00122-024-04809-4

**Published:** 2025-01-11

**Authors:** Renaud Rincent, Junita Solin, Alizarine Lorenzi, Laura Nunes, Yves Griveau, Ludivine Pirus, Dominique Kermarrec, Cyril Bauland, Matthieu Reymond, Laurence Moreau

**Affiliations:** 1 Université Paris-Saclay, INRAE, CNRS, AgroParisTech, GQE - Le Moulon, 91190 Gif-sur-Yvette, France; 2 DELTA Gee, Ajaccio, France; 3 Institut Jean-Pierre Bourgin (IJPB), Université Paris-Saclay, INRAE, AgroParisTech, Versailles, France; 4 INRAE, UE 0972 GCIE, Estrées-Mons, Péronne, France; 5 INRAE, Unité Expérimentale Ressources Génétiques Végétales en Conditions Océaniques (UERGCO), Kéraïber, 29260 Ploudaniel, France

**Keywords:** Phenomic selection, Genomic selection, Maize, Sparse factorial design, Hybrid breeding

## Abstract

**Key message:**

Phenomic selection based on parental spectra can be used to predict GCA and SCA in a sparse factorial design.

**Abstract:**

Prediction approaches such as genomic selection can be game changers in hybrid breeding. They allow predicting the genetic values of hybrids without the need for their physical production. This leads to significant reductions in breeding cycle length, and so to the increase in genetic progress. However, these methods are often underutilized in breeding programs due to the substantial cost involved in genotyping thousands of candidate parental lines annually. To address this limitation, we propose a cost-effective alternative based on phenomic selection, where genotyping of parental lines is replaced by NIR spectroscopy. Standard prediction models are then applied for genomic and phenomic selection, using similarity matrices derived from either genotyping data (genomic selection) or NIR spectral data (phenomic selection). Our hypothesis is that the chemical composition of parental tissues captured by NIRS reflects the genetic similarity between parental lines. We evaluated both strategies using a sparse factorial design, whose hybrids have been phenotyped in a multi-environment trial network, and with NIR spectra acquired on the parental lines on two independent environments. Both genomic and phenomic prediction approaches demonstrated moderate-to-high predictive abilities across various cross-validation scenarios. Our results also showcase the capability of phenomic selection to predict Mendelian sampling. This study serves as a proof of concept that low-cost high-throughput phenomics of parental lines can effectively be used to predict maize hybrids in independent trials. This paves the way for widespread adoption of prediction approaches at the very first stages of hybrid breeding, benefiting both major and orphan species.

**Supplementary Information:**

The online version contains supplementary material available at 10.1007/s00122-024-04809-4.

## Introduction

In plant breeding, an important part of the genetic progress that occurred in the past century was due to our ability to work on replicable genotypes. Observing the same genotype in different microplots and in different environments results in higher heritabilities, which in turn increases genetic progress as illustrated by the breeder’s equation. In maize, working on hybrids obtained by crossing homozygous lines from different heterotic pools is an elegant way of benefiting from the advantage of fixed material while maximizing heterosis. Breeding hybrid species is more resource intensive than autogamous species, because it has to be applied for both parental sides, and because hybrid seeds are costly and time-consuming to produce. Additional constraints are imposed by the number of candidate parental lines available, limiting the feasible creation to only a small subset of all potential hybrids. As a consequence, breeders cross each candidate parental line to only few lines (called testers) of the complementary group, with an important risk of missing excellent crosses. As for any breeding program, phenotyping is the key limiting factor, but it is even more pronounced for hybrid species for this reason.

The use of genome-wide markers to predict the performance of untested genotypes, known as genomic selection (GS, Meuwissen et al. 2001), has opened up new possibilities. The founding work of Bernardo (1994) proved that genomic prediction could be used to predict maize hybrids using genotyping of the parental lines. This means that phenotypic data collected on hybrids can be used to predict and screen hybrids that have never been observed, and even never been produced. The use of molecular markers to estimate kinship between the parental lines allows predicting both the general combining ability (GCA) of any lines and the specific combining ability (SCA) of pairs of lines using as calibration set a very sparse factorial hybrid design, i.e., without the necessity to evaluate several hybrids per line (Seye et al. 2020; Burdo et al. 2021; Lorenzi et al. 2022). This is possible because genomic prediction models work at the marker level, and because different lines can share the same alleles. As a consequence, some researchers have proposed to redesign the usual hybrid breeding scheme based on testcrosses, by a sparse factorial design combined with genomic predictions (Giraud 2016; Fristche-Neto et al. 2018; Burdo et al. 2021; Lorenzi et al. 2022, 2024). By doing so, it would be possible to work directly at the hybrid value level to improve both the GCA and the SCA, avoiding the bias associated with tester choice for GCA estimation and reducing the phenotyping effort for a same number of candidate lines evaluated. So, using a factorial design would allow for a better estimation of GCA and SCA in the early stages of selection. Several studies on the use of factorial designs in early stage selection resulted in good prediction accuracies for untested hybrids (Kadam et al. 2016; Fristche-Neto et al. 2018; Seye et al. 2020; Burdo et al. 2021; Lorenzi et al. 2022) even for a new generation (Lorenzi et al. 2024).

Such an approach based on molecular markers requires the genotyping of all candidate lines that are produced every year. Despite the considerable decrease in the genotyping costs in the last decades, it still consumes considerable amount of money because of the thousands of candidate lines that are produced every year in each breeding program. Frisch et al. (2010) and Fu et al. (2012) have proposed to predict hybrids with gene expression levels measured on the parental lines, instead of genotyping. As transcripts directly capture the expression of the genes and part of the epistasis, they resulted in higher predictive abilities than models based on DNA markers. However, this approach suffers from the same limit as GS, as transcriptomics is still too expensive to be applied on all newly produced inbred lines every year. For this reason, Rincent et al. (2018) have proposed to replace genotyping (or other molecular characterization) by high-throughput phenotyping, and in particular near-infrared (NIR) spectroscopy on grains or other tissues. The key idea is that the absorbance (or reflectance or transmittance) at each wavelength is under polygenic determinism and can thus be used to capture genetic additive and nonadditive similarity between the genotypes. Once the spectra are collected, any genomic prediction model from the frequentist or the Bayesian alphabet or from the field of machine learning (Charmet et al. 2020) can be used with NIR absorbance spectra instead of genotyping data. The main interest is that NIRS is very low cost, or even free because already routinely collected for many species including cereals, legumes, oilseed, or trees (Robert et al. 2022b). This approach, called phenomic selection (PS), was tested on poplar (Rincent et al. 2018), wheat (Krause et al. 2019; Robert et al. 2022a, c), triticale (Zhu et al. 2022), rye (Galán et al. 2020), soybean (Zhu et al. 2021), maize (Lane et al. 2020), grapevine (Brault et al. 2022), rapeseed (Roscher-Ehrig et al. 2024), and coffee (Adunola et al. 2024) with predictive abilities competitive with those obtained with GS. One key point in PS is that once the NIR spectra are collected on the samples, they can be used to make predictions for any polygenic trait in any environment, as long as the calibration set has been phenotyped (Rincent et al. 2018), just like with molecular markers in GS.

PS has been successfully applied on hybrid species (Lane et al. 2020; Adak et al. 2024), but in these studies, the predictions were based on spectra measured on the hybrids. Although promising, this strategy supposes that the hybrids are produced, which considerably reduces its potential value due to the time and cost associated with hybrid production. Here, and similarly to Roscher-Ehrig et al. (2024) on rapeseed, we propose a radically different phenomic selection approaches, in which the performances of the hybrids are predicted using spectral data directly measured on parental lines. This would allow the prediction of hybrids at the very beginning of the breeding programs, before producing the hybrids. If sufficiently accurate, this approach would benefit from the advantages of the sparse factorial design combined with a predictive approach but at much lower cost than GS. To test this, we have compared the predictive abilities of GS and PS in maize based on the genotyping and NIR absorbances of the parental lines of a sparse factorial design between dent and flint lines (Lorenzi et al. 2022). To the best of our knowledge, this is the first study with the objective to predict GCA and SCA using NIR spectra measured on the parental lines. We have considered different cross-validation scenarios with varying levels of relatedness between the training hybrids and the hybrids to be predicted.

## Material and methods

### Genetic material: incomplete factorial design

Originally, six connected biparental dent families and six connected biparental flint families were produced (Giraud et al. 2017a, b; Seye et al. 2020). For each heterotic group, the six families are the result of crosses between four founding lines selected for their silage performance (either silage yield or silage digestibility). The six families were obtained by doubled haploidization (dent) or by five to six generations of selfing using single-seed descent process (flint). The incomplete factorial design is the result of a sparse crossing design between 90 dent lines and 90 flint lines (design F-4H in Lorenzi et al. 2022). In this design, each parental line contributed to produce generally four hybrids. For more details, see Lorenzi et al. (2022). The incomplete factorial design resulted in the production of 363 dent x flint hybrids.

### Phenotyping

The hybrids were evaluated in three trials in 2016 and five in 2017 (Lorenzi et al. 2022). Trials were conducted by INRAE and seven private breeding companies (Lidea, Corteva, Masseeds, KWS, RAGT, Limagrain, Syngenta). The field trials were augmented partially replicated designs (Williams et al. 2011). Two kinds of hybrids were used as controls: two commercial hybrids (RONALDINIO and LG30.275) and 16 founder hybrids that were produced by crossing the four founder lines of each heterotic group. In each trial, controls and 20% of the experimental hybrids were evaluated twice.

Hybrids were evaluated for four agronomic traits (DMY: dry matter yield, DMC: dry matter content, FLOF: female flowering, PH: plant height), and nine silage quality traits (MFU: milk fodder unit, NDF: neutral detergent fiber, DINAG: digestibility of the non-starch and non-soluble carbohydrates, DINAGZ: digestibility of the non-starch, non-soluble carbohydrates, and non-crude protein, LIGN: lignin content, CELL: cellulose content, HCELL: hemicellulose content). Note that those quality traits were predicted with NIRS.

Data curation, computation of adjusted means, and broad-sense heritabilities at the level of the multi-environment trial are presented in detail in Lorenzi et al. (2022). Broad-sense heritabilities were 0.89 for DMY, 0.93 for DMC and FLOF, and 0.86 for MFU. See Lorenzi et al. (2022) for the full variance decompositions.

Correlation between traits was investigated by computing Pearson correlation between adjusted means for each pair of traits.

### Genotyping and NIR spectroscopy

All founder and parental lines were genotyped for 18,480 SNP using an Affymetrix® array provided by Limagrain. Markers with more than 20% missing values within the dent and flint parental lines or with more than 5% (10%) of heterozygosity among the dent (flint) parental lines and markers with minor allele frequency below 5% were removed. Eventually, 9,548 SNP polymorphic (in at least the dent or flint population) were conserved and mapped on a consensus map (Giraud et al. 2017b). Genotypes were coded 0, 0.5, and 1 corresponding to the allele count of the arbitrarily chosen reference allele. Missing genotyping data were imputed as the average allele frequency for the corresponding marker.

NIR spectra were measured on material sampled at two two-row silage trials in 2023 (Ploudaniel and Estrées-Mons, France). Note that these trials are completely independent from the hybrid multi-environment trials (other locations and years). In these two trials were present 71 flint and 65 dent parental lines, the eight founding lines, and the 16 dent x flint hybrids obtained by crossing the 8 founding lines. The trials were composed of two blocks (dent and flint). The eight founder lines and four hybrids obtained by crosses between the founder lines were present in each block. 62% of the dent parental lines and 50% of the flint parental lines were replicated in two microplots in the corresponding block. The genotyping and NIR spectra of the 71 flint and 65 dent parental lines allowed us to compare GS and PS predictive abilities on 222 hybrids of the sparse factorial design.

For each trial, the last ligulated leaf of one plant of each row of each microplot was sampled on the same date around flowering time. These leaves were disposed in a press so that they remained flat, and oven dried at 50 °C during 72 h. NIR spectra were measured at one spot on each side of the midrib on the upper side of each dried leaf with a Fieldspec 2500© portable near-infrared spectrometer (Analytical Spectral Devices, Inc. (ASD), Boulder, CO, USA), used with a bifurcated probe. The spectrum of the light source was measured with a Spectralon ® plate. A Spectralon ® plate was also placed below the leaf while measuring spectra. The spectral range of the spectrometer was 350–2500 nm and its spectral resolution varied from 3 nm for the 350–1000 nm region to 10 nm for the 1000–2500 nm region. The sampling interval was 1.4 nm for the 350–1000 nm region and 2 nm for the 1000–2500 nm region. Each leaf sample was positioned such that the adaxial surface was in contact with light source and fiber optic that leads to the detector.

The two field trials were harvested at silage stage to measure aboveground plant biomass. NIRS measurements were obtained from the harvest of these two trials. A sample of silage harvest was finely grounded and oven dried at 45 °C during 72 h for each microplot. For each microplot, a 2-mL sample of powder was used to measure NIR absorbance with an Antaris II Thermo Fisher Scientific Inc. (Fourier Transform NIRS) between 10,000 cm^−1^ and 4,000 cm^−1^ with a resolution of 4 cm^−1^. For the Estrées-Mons trial, the flint block was not harvested because it was overgrown with weeds.

For both trials (Estrées-Mons and Ploudaniel), both tissues (leaves and whole plant powder), and each wavelength, a smoothing splines spatial model (R package SpATS, (Xose Rodriguez-Alvarez et al. 2018) was fitted with row and column coordinates as random effects, and the genotype as fixed or random effect to compute wavelength adjusted marginal means and broad-sense heritability, respectively. The adjusted means were then normalized (centered and scaled), and their first derivative was computed using a Savitzky–Golay filter (Savitzky and Golay 1964) with a window size of 37 data points and a filter order of 2, implemented in the R package “signal” (Signal Developers 2014).

### Computation of the covariance matrices

Genomic kinship matrices for the dent and flint GCA (${K}_{{GCA}_{d}}$ and ${K}_{{GCA}_{f}}$) were computed from SNP genotypes for all parental lines following the method 1 of (VanRaden 2008) adapted to inbred lines. The kinship coefficient of the dent GCA between individuals i and i’ was estimated as:

$$K_{{GCA_{d} \left( {i,i^{\prime } } \right)}} = { }\frac{{\mathop \sum \nolimits_{m = 1}^{M} \left( {G_{im} - f_{m} } \right)\left( {G_{{i^{\prime } m}} - f_{m} } \right)}}{{\mathop \sum \nolimits_{m = 1}^{M} f_{m} \left( {1 - f_{m} } \right)}},$$

With ${G}_{im}$ the genotype of the dent line I at locus m (coded 0, 0.5, and 1 for BB, BA, and AA genotypes) and ${f}_{m}$ the allele frequency of the reference allele A at locus *m* estimated on the dent parental lines. The kinship matrix ${{\varvec{K}}}_{{{\varvec{G}}{\varvec{C}}{\varvec{A}}}_{{\varvec{f}}}}$ for the flint parental lines was computed similarly. The coefficient of the SCA kinship matrix (${{\varvec{K}}}_{{\varvec{S}}{\varvec{C}}{\varvec{A}}}$) between two dent x flint hybrids, obtained by crossing parental lines i to j and parental lines i’ to j’ was computed as follows (Stuber and Cockerham 1966; Bernardo 1994):

$$K_{{SCA_{{\left( {ij,i^{\prime } j^{\prime } } \right)}} }} = K_{{GCA_{d} \left( {i,i^{\prime } } \right)}} {* }K_{{GCA_{f} \left( {j,j^{\prime } } \right)}} .$$

For NIR spectra, the covariance matrices, also called H matrices in the literature, was computed as in (Robert et al. 2022a):

$H= \frac{{S}_{p}^{*}{S}_{p}^{*{\prime}}}{L}$, with ${{\varvec{S}}}_{{\varvec{p}}}^{\boldsymbol{*}}$ the centered and scaled matrix of pretreated (first derivative of the normalized spectra) spectra, and L the number of wavelengths. This formula was applied on the dent and flint parental spectra separately, resulting in matrices ${H}_{{GCA}_{d}}$ and ${H}_{{GCA}_{f}}$. The coefficient of the SCA spectral covariance matrix (${{\varvec{H}}}_{{\varvec{S}}{\varvec{C}}{\varvec{A}}}$) was computed as:

$$H_{{SCA_{{\left( {ij,i^{\prime } j^{\prime } } \right)}} }} = H_{{GCA_{d} \left( {i,i^{\prime } } \right)}} {* }H_{{GCA_{f} \left( {j,j^{\prime } } \right).}}$$

All genomic and spectral covariance matrices were scaled to have a sample variance of 1 to avoid biased parameter estimations due to different scaling (Kang et al 2010).

### Genomic heritability of the spectra

To evaluate the importance of the genetic variability in the different spectra, we estimated the genomic and the genomic × trial interactions for each wavelength using the following model:

$$y= \left[\begin{array}{c}{y}_{1}\\ {y}_{2}\end{array}\right]= X\beta +Zu+e$$, where **y**_**1**_ and **y**_**2**_ are the phenotypic values (absorbance for a given wavelength) in each trial, **β** is a vector of fixed trial effects, **u** is a vector of random polygenic effects with $$var\left(u\right)= \left(\begin{array}{cc}{\sigma }_{{u}_{1}}^{2}& {\sigma }_{{u}_{12}}\\ {\sigma }_{{u}_{12}}& {\sigma }_{{u}_{2}}^{2}\end{array}\right)\otimes K,$$ e is a vector of independent and normally distributed residuals with$$var\left(e\right)= \left(\begin{array}{cc}{\sigma }_{{e}_{1}}^{2}& 0\\ 0& {\sigma }_{{e}_{2}}^{2}\end{array}\right)\otimes I$$. *I*, *X*, and *Z* are design matrices relating observations to the effects. K is the genomic kinship matrix between dent or flint parental lines as defined above. Following Yamada et al. (1988), the variance/covariance estimates from the previously defined bivariate mixed-model were used to compute estimates of genetic (${\sigma }_{G}^{2}$), genetic by environment (${\sigma }_{GxE}^{2}$) and residual (${\sigma }_{e}^{2}$) variances across sites as follows:${\sigma }_{G}^{2}={\sigma }_{{u}_{12}}$,${\sigma }_{GxE}^{2}= \frac{1}{2}\left({\sigma }_{{u}_{1}}^{2}+ {\sigma }_{{u}_{2}}^{2}\right)-{\upsigma }_{{\text{u}}_{12}}$, and${\sigma }_{e}^{2}=\frac{1}{2}({\sigma }_{{e}_{1}}^{2}+ {\sigma }_{{e}_{2}}^{2})$. We fitted this model on the leaf spectra from both trials separately for the dent parents and the flint parents. For silage of the flint parents, as no spectra were acquired in Mons trial, we implemented a simple univariate G-BLUP model on the spectra obtained on the Ploudaniel silage.

### Pedigree, genomic, and phenomic prediction models

Different prediction models relying on pedigree, genomic, and/or phenomic similarity matrices were defined to compare the predictive abilities of the different predictors and their potential complementarities. These models are:

1 $$\text{P}-\text{BLUP }:Y= {1}_{n}.\mu + {Z}_{d}{p}_{{GCA}_{d}}+{Z}_{f}{p}_{{GCA}_{f}}+{Z}_{df}{p}_{{SCA}_{df}}+\varepsilon ,$$

2 $$\text{G}-\text{BLUP}:Y= {1}_{n}.\mu + {Z}_{d}{g}_{{GCA}_{d}}+{Z}_{f}{g}_{{GCA}_{f}}+{Z}_{df}{g}_{{SCA}_{df}}+\varepsilon ,$$

3 $$\text{H}-\text{BLUP}:Y= {1}_{n}.\mu + {Z}_{d}{h}_{{GCA}_{d}}+{Z}_{f}{h}_{{GCA}_{f}}+{Z}_{df}{h}_{{SCA}_{df}}+\varepsilon ,$$

***Y*** being the vector of phenotype adjusted means across the 11 hybrid trials, $\mu$ the intercept, ${p}_{{GCA}_{d}}$ (${p}_{{GCA}_{f}}$) the random pedigree GCA effect for the dent (flint) parents, with ${p}_{{GCA}_{d}}\sim N(O,{P}_{{GCA}_{d}}{\sigma }_{Pd}^{2})$ and ${p}_{{GCA}_{f}}\sim N(O,{P}_{{GCA}_{f}}{\sigma }_{Pf}^{2})$. ${p}_{{SCA}_{df}}$ is the random pedigree SCA effect with ${p}_{{SCA}_{df}}\sim N(O,{P}_{{SCA}_{df}}{\sigma }_{Pdf}^{2})$. The pedigree relationship matrices between the parental lines were obtained from Lorenzi et al. (2022). Similarly, ${g}_{{GCA}_{d}}$ (${g}_{{GCA}_{f}}$) is the random genomic GCA effect for the dent (flint) parents, with ${g}_{{GCA}_{d}}\sim N(O,{K}_{{GCA}_{d}}{\sigma }_{Gd}^{2})$ and ${g}_{{GCA}_{f}}\sim N(O,{K}_{{GCA}_{f}}{\sigma }_{Gf}^{2})$. ${g}_{{SCA}_{df}}$ is the random genomic SCA effect with ${g}_{{SCA}_{df}}\sim N(O,{K}_{{SCA}_{df}}{\sigma }_{Gdf}^{2})$. ${{\varvec{Z}}}_{{\varvec{d}}}$, ${{\varvec{Z}}}_{{\varvec{f}}}$, and ${{\varvec{Z}}}_{{\varvec{d}}{\varvec{f}}}$ are the corresponding design matrices. $\varepsilon$ is the error, with $\epsilon \sim N(O,I{\sigma }_{\varepsilon }^{2})$, ***I*** being the identity matrix.

Similarly, ${h}_{{GCA}_{d}}$ (${h}_{{GCA}_{f}}$) is the random spectral GCA effect for the dent (flint) parents, with ${h}_{{GCA}_{d}}\sim N(O,{H}_{{GCA}_{d}}{\sigma }_{Hd}^{2})$ and ${h}_{{GCA}_{f}}\sim N(O,{H}_{{GCA}_{f}}{\sigma }_{Hf}^{2})$. ${h}_{{SCA}_{df}}$ is the random hyperspectral SCA effect with ${h}_{{SCA}_{df}}\sim N(O,{H}_{{SCA}_{df}}{\sigma }_{Hdf}^{2})$. Note that we used different spectra origins (tissues and trials) to estimate the hyperspectral similarity matrix, resulting in different H-BLUP models: H.PLOU.LEAF, H.PLOU.SIL, H.MONS.LEAF, and H.MONS.SIL. For example, H.PLOU.LEAF refers to the H-BLUP model that used spectra-based relationship matrices from the NIRS collected on leaves in Ploudaniel. We named H.PLOU.SIL for the model with spectra information measured on the silage powders from Ploudaniel, and so H.MONS.LEAF and H.MONS.SIL follow accordingly. Note that the latter model consists of only a single random effect for the dent GCA as it was not feasible to collect the flint spectra from the trial.

We also combined the different NIRS data in one model:

$$\begin{aligned}\text{H}.\text{ COMB}.&\text{ LEAF}: Y= {1}_{n}.\mu + {Z}_{d}{{h}_{1}}_{{GCA}_{d}}+{Z}_{f}{{h}_{1}}_{{GCA}_{f}}\\&+{Z}_{df}{{h}_{1}}_{{SCA}_{df}}+ {Z}_{d}{{h}_{2}}_{{GCA}_{d}}+{Z}_{f}{{h}_{2}}_{{GCA}_{f}}\\&+{Z}_{df}{{h}_{2}}_{{SCA}_{df}}+\varepsilon\end{aligned}$$

$$\begin{aligned}\text{H}.\text{ COMB}&.\text{ SIL}:Y= {1}_{n}.\mu + {Z}_{d}{{h}_{3}}_{{GCA}_{d}}+{Z}_{f}{{h}_{3}}_{{GCA}_{f}}\\&+{Z}_{df}{{h}_{3}}_{{SCA}_{df}}+{Z}_{d}{{h}_{4}}_{{GCA}_{d}}+ \varepsilon\end{aligned}$$

$$\begin{aligned}\text{H}.\text{ ALL}:&Y= {1}_{n}.\mu + {Z}_{d}{{h}_{1}}_{{GCA}_{d}}+{Z}_{f}{{h}_{1}}_{{GCA}_{f}}\\&+{Z}_{df}{{h}_{1}}_{{SCA}_{df}}+ {Z}_{d}{{h}_{2}}_{{GCA}_{d}}+{Z}_{f}{{h}_{2}}_{{GCA}_{f}}\\&+{Z}_{df}{{h}_{2}}_{{SCA}_{df}}+{Z}_{d}{{h}_{3}}_{{GCA}_{d}}+{Z}_{f}{{h}_{3}}_{{GCA}_{f}}\\&+{Z}_{df}{{h}_{3}}_{{SCA}_{df}}+ {Z}_{d}{{h}_{4}}_{{GCA}_{d}}+ \varepsilon\end{aligned}$$

H.COMB.LEAF has six random effects structured for the GCA and SCA effects of which each effect has a variance–covariance structure derived from the leaves spectra in Ploudaniel (h_1_) and Estrées-Mons (h_2_). Similarly, we used the silage spectra in Ploudaniel (h_3_) and Estrées-Mons (h_4_) in the H.COMB.SIL model. It has only one random effect of SCA (h_3_) due to the absence of flint silage spectra in Mons. H.ALL is the model with combined leaves and silage spectra, which could be seen as adding the effects from H.COMB.LEAF and H.COMB.SIL models.

### Prediction scenarios and the corresponding cross-validation schemes

We compared the predictive abilities of the different pedigree, genomic, and phenomic prediction models in different prediction scenarios.

The first prediction scenario (CV_SparseTesting) was a simple fivefold cross-validation with five repetitions for each trait. In this scenario, the fivefold partitions were determined by randomly sampling among the 222 hybrids, i.e., not considering population structure within the parental lines.

In the second scenario (CV_newDentFlint), we considered the fact that the dent parents (and the flint parents) are clustered into six families. In this scenario, the partitions between the calibration set and predicted set were determined in such a way that the dent and flint parents of the predicted hybrids were from different families than the parents of the hybrids composing the calibration set. In this scenario, we considered only the four calibration set/prediction set partitions for which there were at least 10 hybrids in the predicted set. In this scenario, the pedigree relationship to the calibration hybrids was the same for all the predicted hybrids. This means that no prediction based on pedigree was possible. This scenario was designed to determine if phenomic selection was able to predict beyond pedigree relationships.

For all prediction scenarios, all models were applied exactly on the same partitions for a fair comparison. Predictive abilities were computed for each fold as the Pearson correlation between the predictions and the adjusted means of the predicted set of varieties.

## Results

### Correlation between traits

**Fig. 1:**
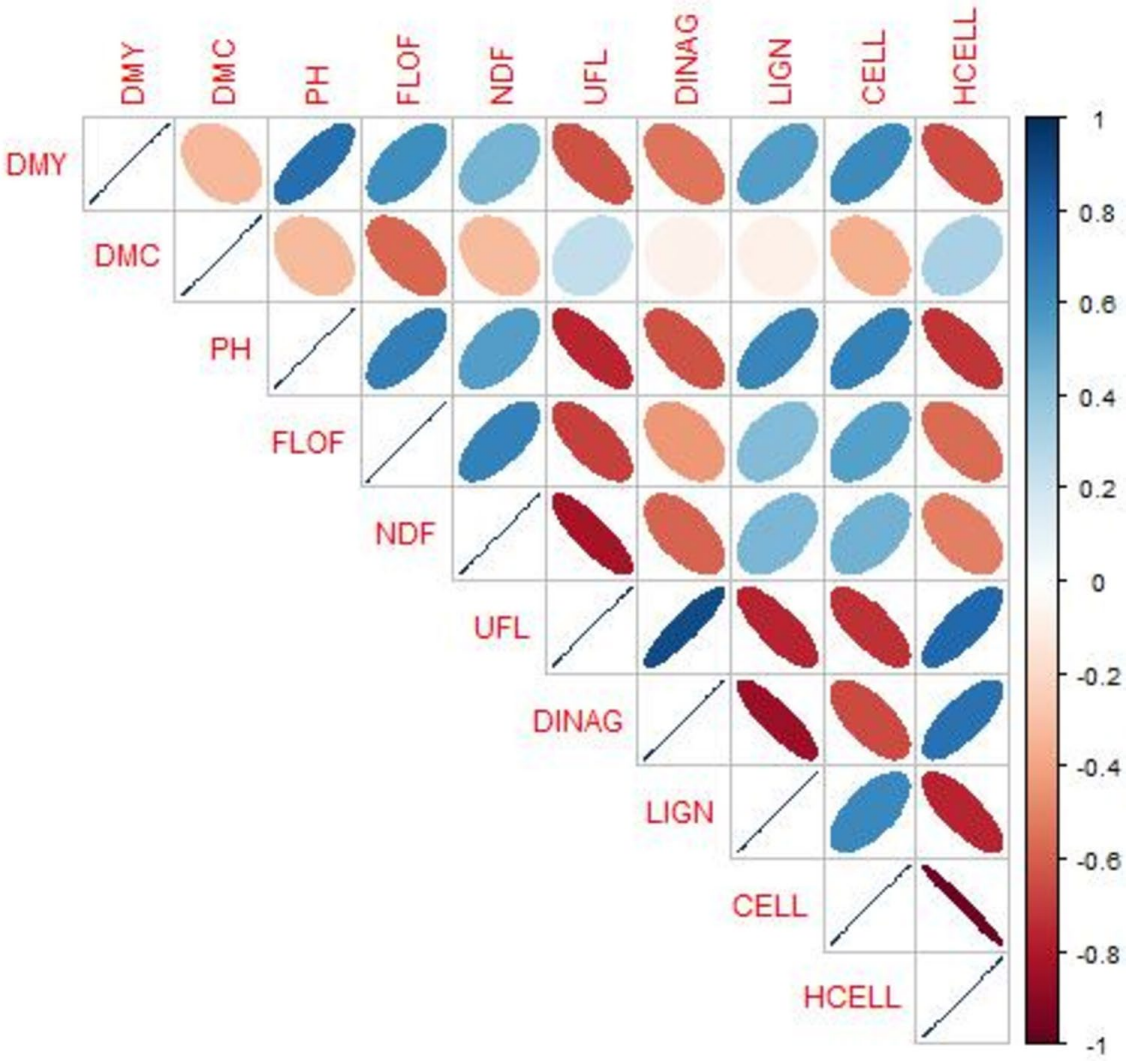
Correlation plot between each pair of traits (Pearson correlations)

Correlations between traits were highly variable, and were particularly strong (positively or negatively) between pairs of quality traits (Fig. 1), such as between UFL and DINAG (0.89), and UFL and NDF (− 0.83). There were also strong correlations between agronomic traits and quality traits, for instance between PH and UFL (− 0.75) or HCELL (− 0.72), or between DMY and UFL (− 0.64) or HCELL (− 0.65). Correlations between agronomic traits could also be strong, for instance between PH and FLOF (0.68), or between DMY and PH (0.75).

### Genomic heritability of the spectra

**Fig. 2:**
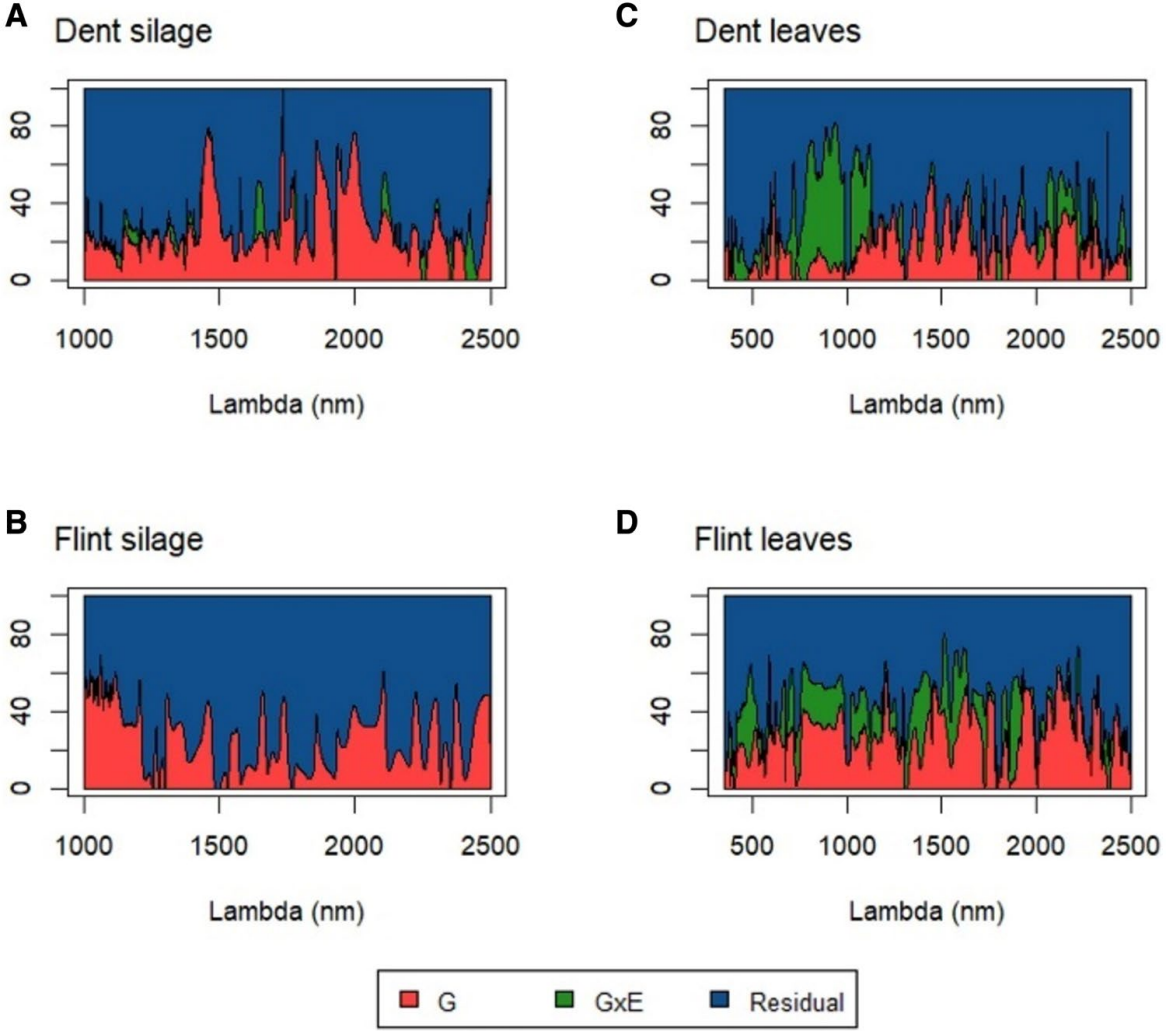
The proportion of genetic (red), genetic-environment interaction (green), and residual (blue) of the spectra variance measured in **A** dent silage, **B** flint silage in Ploudaniel, **C** dent leaves, and **D** flint leaves. NIRS analysis from leaves and dent silage was performed for two locations: Ploudaniel and Estrées-Mons. The variance decompositions were obtained from a bivariate model if spectra were acquired in the two trials or with a univariate model if the spectra were obtained only in Ploudaniel (flint silage) (color figure online)

We assessed genomic heritability by decomposing the variance of absorbance along the wavelength into genetic, genetic by environment (for the leaves spectra only), and residual variance. Genomic heritability appeared to be highly variable along the spectra taking values between 0 and close to 100% (Fig. 2). On average, the spectra from silage (average heritability of 25.3%) were more heritable than those from the leaves (19.0%). Interestingly, among the two groups of genotypes (i.e., dent parents and flint parents), spectra measured on the flint parents have a stronger signal related to genetics. (The average percentage is 28.0%.) The shapes of the genomic heritability scans also highlight the strong and variable genotype-environment interactions along the wavelengths, ranging from 0 to 73.7%.

### Predictive abilities in the sparse testing scenario (CV_SparseTesting)

**Fig. 3:**
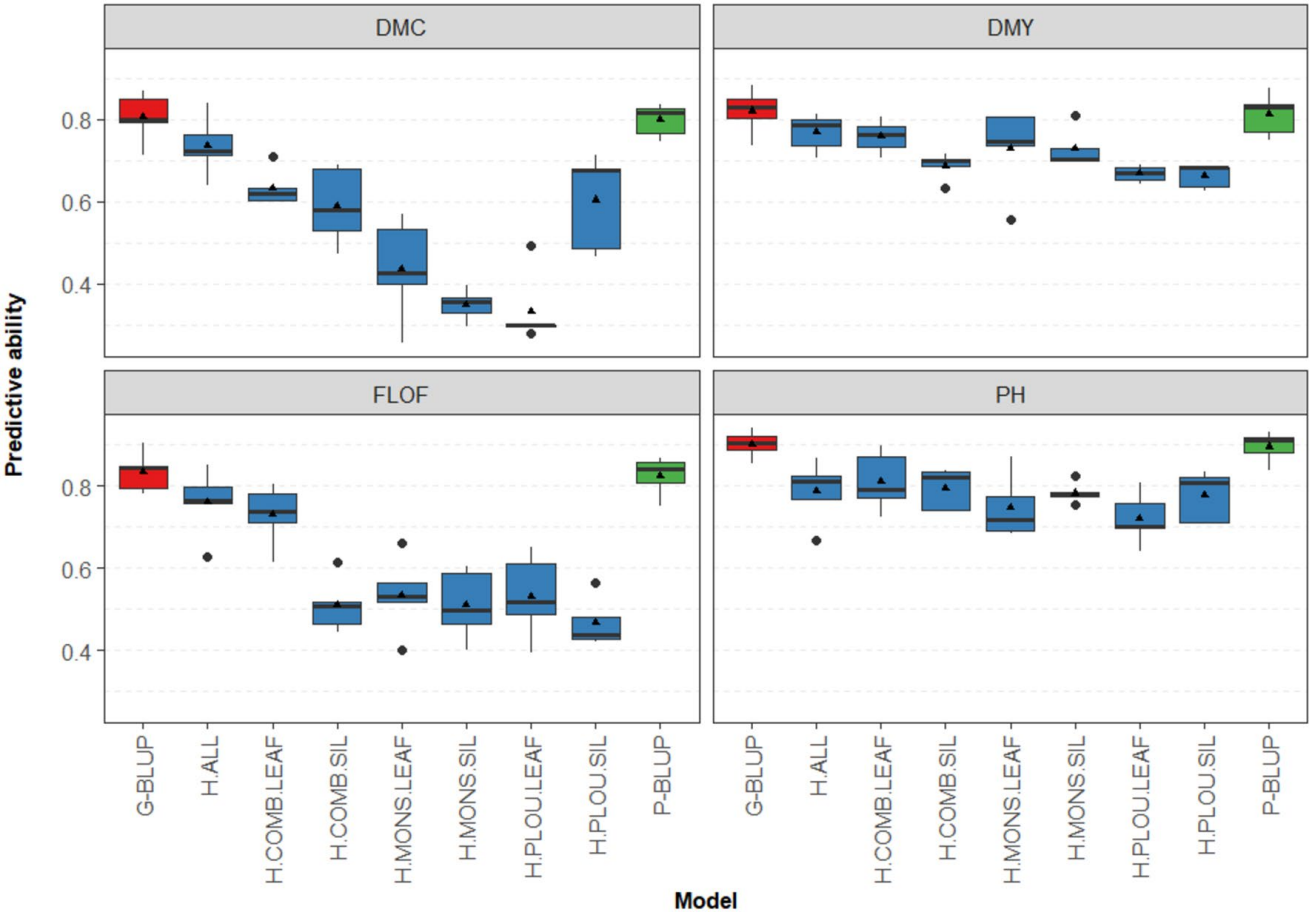
Predictive abilities of the pedigree, genomic, and phenomic prediction models for the agronomic traits in the CV_SparseTesting scenario. These were fivefold random cross validations with 5 repeats. The boxes represent the medians, as well as the first and third quartiles and the triangle dots marked the mean. Each plot was labeled corresponding to a given trait of which DMC: Dry Matter Content, DMY: Dry Matter Yield, FLOF: Flowering Time, and PH: Plant Height

The average predictive abilities for scenario CV_SparseTesting were high with models P-BLUP (ranging from 0.66 to 0.90) and G-BLUP (ranging from 0.70 to 0.90) for all agronomic (Fig. 3) and quality traits (Supplementary Fig. S1), although these values are quite variable between traits. For example, plant height was predicted with a mean predictive ability of 0.90 for both P-BLUP and G-BLUP (Fig. 3), while neutral detergent fiber content (NDF, Supplementary Fig. S1) was less predictable with a mean predictive ability of 0.66 with P-BLUP and 0.70 with G-BLUP.

The predictive abilities of the PS models for agronomic traits were moderate to high, ranging from 0.33 (H.PLOU.LEAF) to 0.79 (H.ALL), implying that the prediction accuracy depends on the spectra that were used. The best PS model is the one with combined spectra from the two tissues from the two trials. Similar to G-BLUP and P-BLUP, predictive abilities are also highly variable depending on the trait, for instance the mean values range narrowly from 0.66 to 0.77 for DMY predictions, yet mean predictive abilities for predicting FLOF range from 0.47 to 0.76 (Fig. 3).

The predictive abilities obtained with the best PS model were slightly lower than those obtained with GS, for instance, they ranged from 0.71 to 0.81 in predicting DMY while GS gave accuracies from 0.74 to 0.88. For the four agronomic traits, models based on leaf spectra from both locations appeared to be more accurate than models based on silage spectra (0.71–0.80 against 0.63–0.71 for DMY predictions, Fig. 3), although this trend is not necessarily visible for single spectrum models.

### Predictive abilities in the CV_newDentFlint scenario

When predicting hybrids that were genetically distant to the training set, the predictive abilities were as expected much lower than in the previous scenario. In this scenario (CV_newDentFlint), it was not possible to make predictions based on pedigree information, as the predicted hybrids all had the same pedigree coefficients with the training hybrids.

**Fig. 4:**
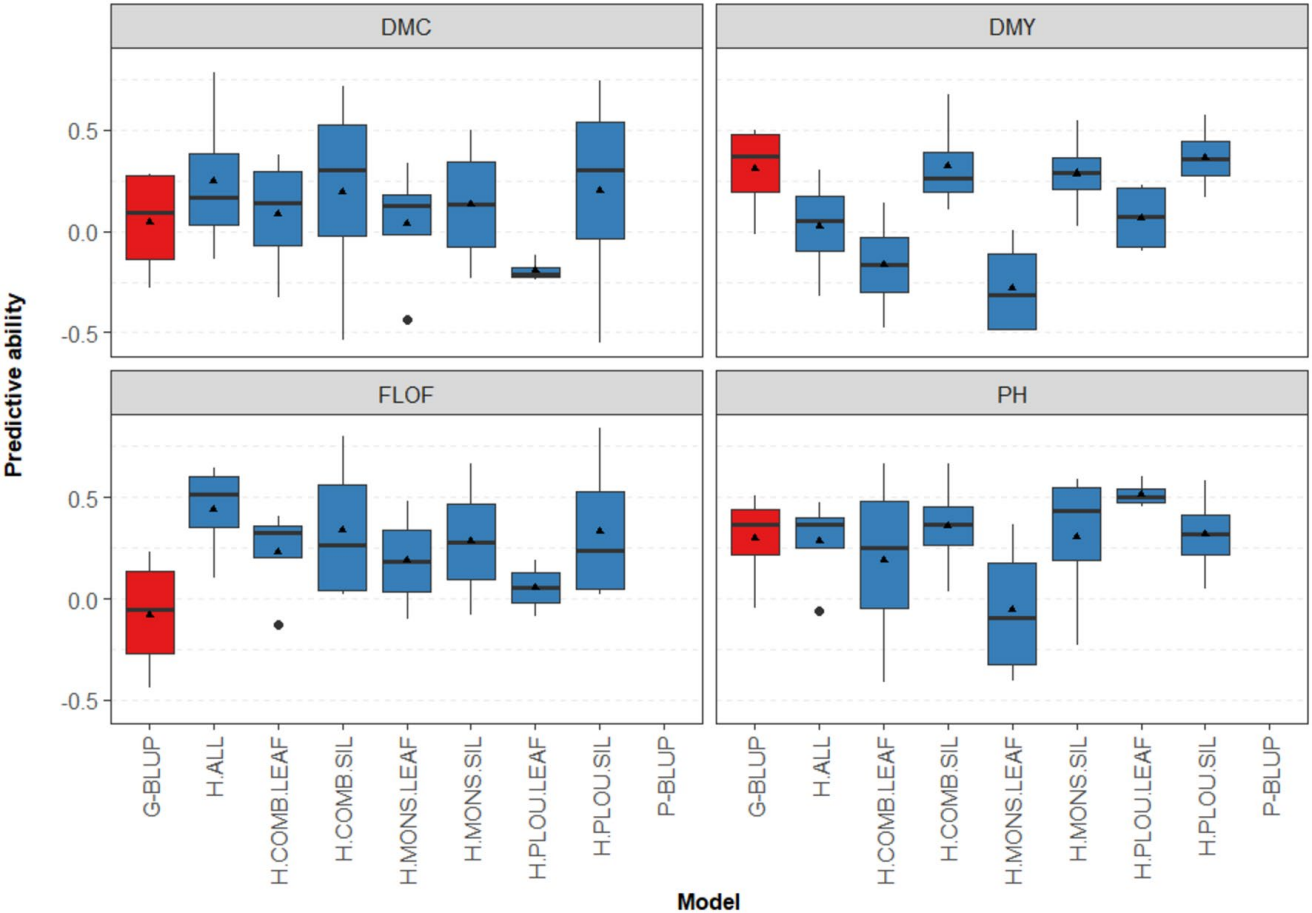
Predictive abilities for the CV_newDentFlint scenario for all traits. Only the four calibration set/prediction set partitions with more than 10 hybrids in the prediction set were considered. The boxes represent the median, as well as the first and third quartiles and the triangle dots marked the mean. There is no boxplot for P-BLUP on this figure, as this model cannot be used for this scenario, in which the pedigree coefficient is the same for all the predicted hybrids

The predictive abilities of G-BLUP varied with a mean between −0.08 and 0.41 (Figs. 4, S2). Again, the predictive abilities of H-BLUP were highly variable depending on the tissues and trials used to measure the spectra. In general, the best H-BLUP model was the one using all the NIRS data. Contrary to the previous scenario, this model (H.ALL) was able to perform similarly or better than the G-BLUP model for most traits, except for DMY and PH (Table 1), though another PS model (H.PLOU.SIL) outperformed G-BLUP for both traits.

**Table 1:** Average predictive abilities for the four agronomic traits under the CV_newDentFlint scenario. DMY: Dry Matter Yield, DMC: Dry Matter Content, PH: Plant Height, FLOF: female flowering. P-BLUP is a pedigree-BLUP model, and G-BLUP is a genomic-BLUP model. Models starting with “H” are phenomic prediction models based on NIR spectra measured on leaves (”LEAF”) or silage (“SIL”) from plants grown in Ploudaniel (“PLOU”) or Estrées-Mons (“MONS”). We combined the spectra of the two locations (“COMB”) or the spectra of both locations and tissues (“H.ALL”)

	DMY	DMC	PH	FLOF
P-BLUP	NA	NA	NA	NA
G-BLUP	0.31	0.05	0.30	−0.08
H.PLOU.LEAF	0.07	− 0.19	0.52	0.05
H.MONS.LEAF	− 0.28	0.04	−0.06	0.19
H.PLOU.SIL	0.36	0.20	0.32	0.33
H.MONS.SIL	0.29	0.13	0.31	0.28
H.COMB.LEAF	− 0.17	0.08	0.19	0.23
H.COMB.SIL	0.33	0.20	0.36	0.34
H.ALL	0.02	0.24	0.29	0.44

Contrary to the previous scenario, spectra from silage resulted in more accurate predictions for most traits in comparison to the ones from leaves. For example, predictions for DMY have a mean predictive ability of 0.33 with combined silage spectra and −0.17 with combined leaves spectra.

## Discussion

In the phenomic selection founding paper, Rincent et al. (2018) have shown on wheat and poplar that NIRS was able to capture genetic similarity between the genotypes. As a consequence, the spectra could be used to predict any trait, even if completely unrelated to the tissue on which NIR spectra were acquired. Even in the extreme situation in which NIR spectra were measured in one environment to predict traits measured in completely independent environments, PS could result in higher predictive abilities than GS. Since then, PS has been evaluated on other species including maize (Lane et al. 2020; Adak et al. 2024). These studies proved that spectral data acquired on hybrids could result in predictive abilities competitive with GS. However, implementing this PS approach to hybrid breeding programs necessitate to produce all the hybrids that have to be predicted. This is a severe drawback in comparison to GS, in which any potential hybrid can be predicted before being produced as long as the genotyping of the parental lines is known. But recently, Roscher-Ehrig et al. (2024) went a step further and predicted testcrosses with the NIR spectra of the parental lines. They reached high predictive abilities, competitive to those obtained with GS, proving that phenomic predictions could be used to predict GCA without producing the hybrids. Here, we propose a new application of PS, in which spectra measured on the parental lines are used to predict hybrids using both GCA and SCA. We evaluated this strategy using NIR spectra acquired on the parental lines of an incomplete factorial design (Lorenzi et al. 2022).

### NIR spectra are under polygenic determinism

We first estimated the genomic heritability along the spectra for the dent and the flint parents (Fig. 2). We observed a strong variability along the spectra, with regions with a very high heritability and others with no heritability at all. This confirms results obtained in previous studies showing that the amount of genetic signal is highly variable along the spectra (Rincent et al. 2018; Brault et al. 2022). We also observed that the patterns of heritability along the spectra were different between tissues and between locations. These results highlight the fact that the NIR spectra are under polygenic determinism, and under strong genotype x environment interactions, as illustrated in Robert et al. (2022c). The average genomic heritability was comparable in both tissues, meaning that they should be both able to capture and predict polygenic traits.

### Phenomic selection based on parental spectra is competitive with genomic selection

In the CV_SparseTesting scenario, P-BLUP and G-BLUP performed similarly well, highlighting the strong structuration of the datasets due to the fact that the parental lines were obtained from 6 biparental crosses and that each hybrid had on average three half-sibs. The predictive abilities of the best PS model were not far behind, and as high as 0.78 for DMY or 0.73 for DMC (Fig. 3). However, the results were highly variable depending on the origins of the spectra (tissue and location), the best model being the one combining all the spectra, illustrating the complementarity of the different spectra. This probably means that characterizing each genotype by only one spectrum is not sufficient to saturate the phenotypic space, and that combining spectra measured at different dates, on different tissues or in different locations (NIR or other high-throughput measurements) should be particularly useful to increase predictive abilities. Nevertheless, we see that even spectra acquired on only one tissue and in one location could result in high predictive abilities (e.g., 0.73 for DMY with the spectra measured on leaves in Mons, Fig. 3).

### NIR spectra are able to capture Mendelian sampling (scenario CV_newDentFlint)

In situations in which the expected relatedness coefficient between the training set and the predicted set is equal for all the predicted hybrids, it is not possible to make predictions with the pedigree relationship matrix (model P-BLUP). In this scenario (CV_newDentFlint), we however observed that phenomic selection was able to predict the new hybrids with average predictive abilities of 0.02–0.50, competitive with those obtained with G-BLUP (−0.08 to 0.41, Figs. 4 and S2). In this scenario, for all the agronomic traits, there was always at least one type of spectra for which PS was more accurate than GS. These results prove that the spectra are able to capture Mendelian sampling, meaning that they can be used to predict within biparental populations. In this scenario, NIR spectra acquired on silage were more efficient than those measured on the leaves. One explanation could be that the silage spectra of the parental lines were quite efficient to predict some of the quality traits on hybrids (supp. Table S1), which were in turn strongly correlated to the agronomic traits (Fig. 1). It was indeed shown that silage quality of hybrids can be strongly correlated with the silage quality of the parental lines (Argillier et al. 2000). This suggests that spectra taken on inbred lines at silage stage could be used to predict silage quality traits of hybrids. However, note that the comparison of the efficiency of the two kinds of tissue (leaf and silage) is not straightforward, as different spectrometers were used depending on the tissues. The surprising low performance of the G-BLUP model in this situation might be explained by (i) the fact that hybrids between lines from the parental lines families of the predicted hybrids were excluded from the calibration set and (ii) the low number of parental lines contributed to the hybrids of the calibration set. The low performance of across family prediction in multiparental populations especially with low calibration sizes is consistent with results from Zhao et al. 2012; Riedelsheimer et al. 2013 or Lehermeier et al. 2014. Zhao et al. 2012 suggested that such results could be due to epistatic effects not captured by genomic prediction models. Phenomic predictions might have captured such epistatic effects at the inbred lines levels that might have contributed to the predictions at the hybrids level.

### Implementation of phenomic selection in hybrid programs

As in Robert et al. (2022a), we observed an important effect of the origin of the spectra on the predictive abilities. Both the tissue and the environment of origin had a strong effect on predictive abilities, and the best spectra were not always the same depending on the predicted trait. However, we generally observed that aggregating different spectra resulted in higher predictive abilities. This probably means that a single spectrum is not sufficiently rich to fully characterize a genotype. There is a clear necessity to study and optimize the technical aspects of NIR acquisition and there seems to be considerable room for improvement. All the aspects related to the choice of the spectrometer, the number of wavelengths, the tissue, the number of spectra per genotype, and the combination with drone measurements, need to be optimized for an efficient use of phenomic selection (Robert et al. 2022b).

One important conclusion from our study is that we were able to predict with high predictive abilities hybrids with spectra measured on the parental lines in independent trials. This opens the way to new applications of phenomic selection for hybrid breeding programs. It is currently acknowledged that the most efficient use of predictive approaches is to predict as early as possible in the breeding program to eliminate the poor performing individuals. At these steps, more than 90% of the genotypes are eliminated without any information on productivity. This means that having predictions of GCA and SCA for productivity on these genotypes, even with low predictive abilities, would be a game changer as it would allow to remove an important part of the genotypes without losing the best ones. This is currently not possible with genomic selection because of the cost of genotyping, but we could demonstrate here that this strategy could be envisaged with phenomic selection even for hybrid species.

## Supplementary Information

Below is the link to the electronic supplementary material.

Supplementary file1 (DOCX 175 KB)

## Data Availability

The datasets generated during and/or analyzed during the current study are available from the corresponding author on reasonable request.
