# Supplementary material for: Using phenomic selection to predict hybrid values with NIR spectra measured on the parental lines: proof of concept on maize
Source: Theor Appl Genet. 2025 Jan 11;138(1):28. doi: 10.1007/s00122-024-04809-4 (PMC11724800; doi:10.1007/s00122-024-04809-4)
Supplement: Supplementary file 1 — Supplementary file1 (DOCX 175 KB) [file 122_2024_4809_MOESM1_ESM.docx]

**Supplementary figures and tables**

Table S1. Average predictive abilities for the seven agronomic traits under the CV_newDentFlint scenario. NDF: neutral detergent fiber content, MFU: milk fodder unit per kilogram of dry matter, DINAG and DINAGZ: cell wall in-vitro digestibility criteria, CELL: cellulose content in the cell wall, HCELL: hemicellulose content, LIGN: lignin content.

|  | NDF | MFU | DINAG | DINAGZ | LIGN | CELL | HCELL |
| --- | --- | --- | --- | --- | --- | --- | --- |
| P-BLUP | NA | NA | NA | NA | NA | NA | NA |
| G-BLUP | 0.16 | 0.25 | 0.41 | 0.22 | 0.29 | 0.19 | 0.24 |
| H.PLOU.LEAF | -0.07 | 0.00 | -0.09 | -0.20 | 0.14 | 0.18 | 0.23 |
| H.MONS.LEAF | 0.18 | -0.02 | 0.01 | 0.15 | 0.04 | -0.04 | -0.04 |
| H.PLOU.SIL | 0.36 | 0.48 | 0.18 | -0.22 | 0.14 | 0.21 | 0.25 |
| H.MONS.SIL | NA | 0.14 | -0.11 | -0.01 | -0.07 | 0.23 | 0.39 |
| H.COMB.LEAF | 0.13 | 0.05 | 0.02 | 0.07 | 0.13 | 0.05 | 0.11 |
| H.COMB.SIL | 0.35 | 0.48 | 0.18 | -0.20 | 0.14 | 0.23 | 0.27 |
| H.ALL | 0.45 | 0.50 | 0.17 | 0.03 | 0.16 | 0.25 | 0.30 |


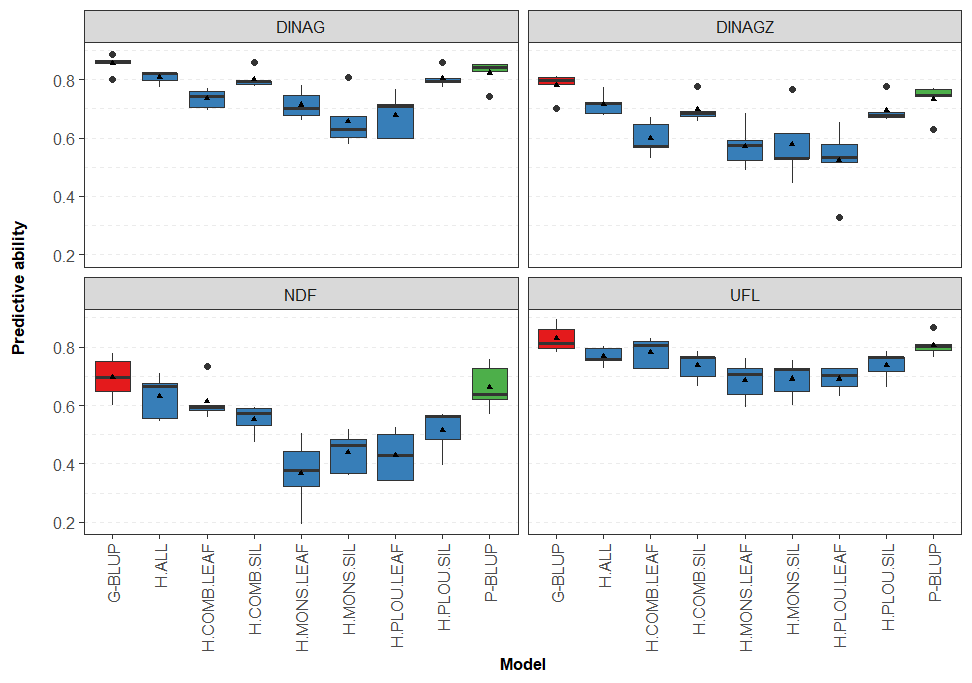

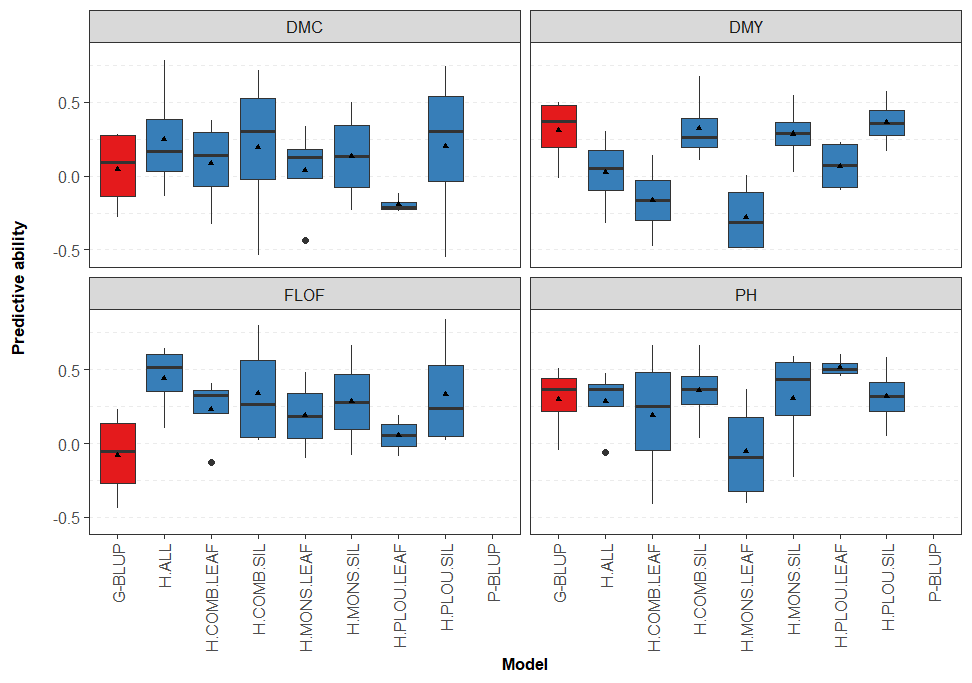


Supplementary figure S1. Predictive abilities for the quality traits under the sparse testing scenario. Each label corresponds to the phenotypic trait, i.e. DINAG and DINAGZ: cell wall in-vitro digestibility criteria, NDF: neutral detergent fiber content, MFU: milk fodder unit per kilogram of dry matter, CELL: cellulose content in the cell wall, HCELL: hemicellulose content, LIGN: lignin content.


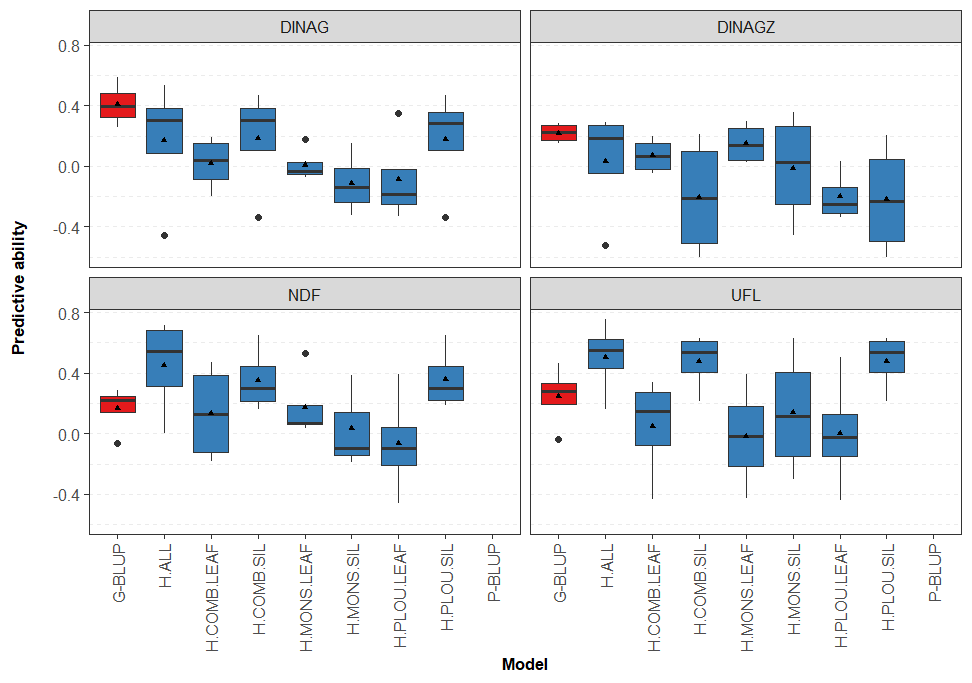

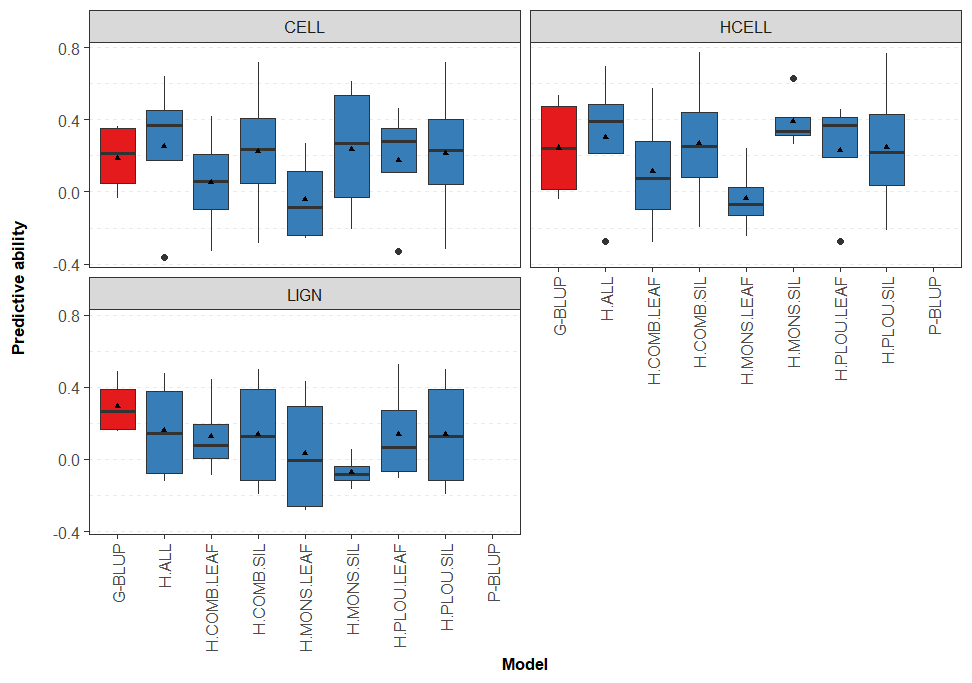


Supplementary figure S2. Predictive abilities for the quality traits under the CV_newDentFlint scenario. Each label corresponds to the phenotypic trait, i.e. DINAG and DINAGZ: cell wall in-vitro digestibility criteria, NDF: neutral detergent fiber content, MFU: milk fodder unit per kilogram of dry matter, CELL: cellulose content in the cell wall, HCELL: hemicellulose content, LIGN: lignin content. There is no boxplot for P-BLUP on this figure, as this model cannot be used for this scenario, in which the pedigree coefficient is the same for all the predicted hybrids.
